# Functional characterization of *Fagopyrum tataricum ZIP* gene family as a metal ion transporter

**DOI:** 10.3389/fpls.2024.1373066

**Published:** 2024-04-12

**Authors:** Xinrong Zhang, Jiao Kong, Lingzhi Yu, Anhu Wang, Yi Yang, Xiaoyi Li, Jianmei Wang

**Affiliations:** ^1^ Key Laboratory of Bio-Resources and Eco-Environment of Ministry of Education, College of Life Sciences, Sichuan University, Chengdu, China; ^2^ Panxi Crops Research and Utilization Key Laboratory of Sichuan Province, Xichang College, Xichang, China

**Keywords:** *Fagopyrum tataricum*, Tartary buckwheat, FtZIPs, ion transportation, yeast heterologous complementation

## Abstract

The zinc/iron-regulated transporter-like proteins (ZIP) family acts as an important transporter for divalent metal cations such as Zn, Fe, Mn, Cu, and even Cd. However, their condition is unclear in Tartary buckwheat (*Fagopyrum tataricum*). Here, 13 ZIP proteins were identified and were predicted to be mostly plasma membrane-localized. The transient expressions of *FtZIP2* and *FtZIP6* in tobacco confirmed the prediction. Multiple sequence alignment analysis of FtZIP proteins revealed that most of them had 8 putative transmembrane (TM) domains and a variable region rich in histidine residues between TM3 and TM4, indicating the reliable affinity to metal ions. Gene expression analysis by qRT-PCR showed that *FtZIP* genes were markedly different in different organs, such as roots, stems, leaves, flowers, fruits and seeds. However, in seedlings, the relative expression of *FtZIP10* was notably induced under the CdCl_2_ treatment, while excessive Zn^2+^, Fe^2+^, Mn^2+^ and Cd^2+^ increased the transcript of *FtZIP5* or *FtZIP13*, in comparison to normal conditions. Complementation of yeast mutants with the FtZIP family genes demonstrate that FtZIP7/10/12 transport Zn, FtZIP5/6/7/9/10/11 transport Fe, FtZIP12 transports Mn and FtZIP2/3/4/7 transport Cd. Our data suggest that FtZIP proteins have conserved functions of transportation of metal ions but with distinct spatial expression levels.

## 1 Introduction

Zinc (Zn), as a vital micronutrient, is essential for the functions of numerous proteins, and physiological processes in prokaryotes and eukaryotes (Mondal et al., 2013). It has been demonstrated that Zn deficiency is one of the most widespread minerals nutritional problems affecting the development and health of plants under field conditions, whereas excessive amounts of Zn inhibit the plant’s growth and development (Briat and Lebrun, 1999). For example, excessive Zn may be highly toxic, and in some cases, may cause damage by the production of harmful reactive oxygen species (Sinclair et al., 2018).

To maintain the intracellular and extracellular metal concentration (Jiang et al., 2021), plant cells have evolved multiform transport networks to balance the absorption, utilization, and storage of trace metal elements, including iron (Fe), manganese (Mn), copper (Cu), and Cadmium (Cd) (Ajeesh Krishna et al., 2020). Studies have demonstrated that HMA (Heavy Metal ATPase) proteins, CDF (Cation-Diffusion Facilitator), and ZIP (Zrt/Irt-like protein family) act as important regulators in these processes (Bari et al., 2021). For example, *AtHMA2* and *AtHMA4* are required for Cd translocation in *Arabidopsis thaliana* (Wong and Cobbett, 2009). A member of the Mn-cation diffusion facilitator (CDF) family, MTP8.1 (METAL-TOLERANCE PROTEIN), plays a central role in high Mn tolerance by sequestering Mn into vacuoles (Tsunemitsu et al., 2018). Overexpression of *HvZIP7* in barley plants would increase Zn uptake (Tiong et al., 2014).

The ZIP family has a major role in Zn transportation and metal homeostasis in planta (Wang et al., 2017). Both ZIP1 and ZIP3 in Arabidopsis are involved in Zn input from soil to root (Grotz and Guerinot, 2006). In grapevines, a deficit of Zn results in abnormal leaves, showing reduced area, mottled and shortened internodes (Gainza-Cortés et al., 2012). The *VvZIP3* is involved in Zn uptake and distribution during the early reproductive development of *Vitis vinifera* (Gainza-Cortés et al., 2012). The expression of *NtZIP4B* from tobacco (*Nicotiana tabacum*) is upregulated by Zn deficiency, however, downregulated by Zn excess (Barabasz et al., 2018). Overexpressing *ZmZIP5* would increase the accumulation of Zn and Fe in the roots and shoots of maize (*Zea mays*), whereas decreases in the seeds (Li S et al., 2019). The expression of *OsZIP4* is upregulated by low Zn and regulates the transportation of Zn to the tiller bud in rice (*Oryza sativa*) (Ishimaru et al., 2005; Mu et al., 2021). In barley (*Hordeum vulgare*), *HvZIP3*, *HvZIP5* and *HvZIP8* act as Zn transporters involved in Zn^2+^ homeostasis, but not Fe or Mn transporter (Pedas et al., 2009).

Besides Zn, ZIP transporters have been reported to regulate the transport other transition metal cations, including Mn, Fe, Cd, Cu, cobalt (Co)and nickel (Ni). For example, *OsZIP9* can take up Zn and Co from external media into root cells (Yang et al., 2020). Interestingly, *OsZIP6* in *Xenopus laevis* oocytes could mediate the uptake of Co, Fe, and Cd but not Zn, Mn, and Ni (Kavitha et al., 2015). Knockout of *OsZIP7* shows retention of Zn and Cd in roots and basal nodes, resulting in the inhibition of their upward delivery to upper tissues (Tan et al., 2019). The *HvIRT1* from *Hordeum vulgare*, with a high similarity to OsIRT1 from *Oryza sativa*, controls Mn uptake in the root (Pedas et al., 2008).

Studies show that there are 15 members of the ZIP family in *A. thaliana* (Grotz et al., 1998), 23 in bean (*Phaseolus vulgaris* L) (Astudillo et al., 2013), 15 in rice (Narayanan et al., 2007), 14 in wheat (*Triticum aestivum)* (Evens et al., 2017) and 12 in maize (Mondal et al., 2013). Although *ZIP* genes have been extensively studied in crops, such as rice, genome-wide analysis of the members of this family has yet to be uncovered in Tartary buckwheat (*Fagopyrum tataricum*). The *F. tataricum* has been widely popularized as a food and ornamental crop in East Asian countries, particularly, in Southwestern China (Li and Zhang, 2001). Buckwheat is widely adaptable to low-fertility soils and some mountainous regions, in particular, exhibiting short growth cycles (Fan et al., 2021; Li et al., 2022). Recently, *F. tataricum* is recognized as a good source of nutritionally valuable proteins, lipids, dietary fibers, minerals, and other health-promoting compounds, such as phenolic and sterols (Noreen et al., 2021; Lin et al., 2023). Thus, it has received increasing attention as a potential functional food. Due to the importance of ZIPs in metal ion absorption, transport and distribution, recent studies have focused on cloning and characterizing their functions in the important plants such as *Arabidopsis thaliana* or rice, as well as major food and horticultural crops. However, limited information is available on ZIPs in Tartary buckwheat. Cloning and functional analysis of Tartary buckwheat ZIPs can significantly promote the understanding of potential metal element absorption mechanisms. Furthermore, the sequencing and assembling of the buckwheat genome provides an opportunity to identify and isolate these genes at the genomic level (Zhang et al., 2017). Here, we used protein and gene structure analysis, phylogenetic analysis, and sequence alignment, to identify ZIP family in *F. tataricum*. We also analyzed the spatial expression including root, stem, leaf, flower, fruits and seed, and also checked the inducible expression of *FtZIP* genes in response to Zn, Cd, Fe and Mn. In addition, the ability of FtZIPs to transport four metal ions was tested by the yeast complementation assay.

## 2 Materials and methods

### 2.1 Plant materials and stress treatments

The Tartary buckwheat cultivar (XiQiao #7) was used in this study. In the indicated stages, we collected tissues of roots, stems, leaves, flowers, fruits and seeds in Tartary buckwheat, frozen in liquid N_2_, and stored at -80°C. For investigating the expressions of *FtZIPs*, after 21 days of growth, the XiQiao #7 seedlings were removed from the flowerpot to avoid damaging the roots. The soil in the roots was meticulously cleaned. The seedlings were treated with Hoagland medium containing different concentrations of metal ions, specifically 100 μM CdCl_2_, 75 μM ZnSO_4_, 100 μM MnCl_2_ or 100 μM FeSO_4_ for 6 h, collected treated seedlings and frozen in liquid N_2_, and stored at -80°C. Seedlings and *Nicotiana benthamiana* plants were grown in a growth chamber under 60% relative humidity and with a day/night cycle of 16 hr light 114/8 hr dark and 120 μmol m^–2^ s^–1^.

### 2.2 Identification and bioinformatics analyses of *FtZIP* genes

The sequence of the Tartary buckwheat proteins was downloaded from the Tartary buckwheat database (TBD, http://www.mbkbase.org/Pinku1/), after which the HMM profile was downloaded from the Pfam protein family database (http://pfam.sanger.ac.uk/). The *ZIP* gene family was searched by BLASTP methods. HMMER3.1 was used to search against the buckwheat protein sequence with a threshold of E < 1e^-5^ (Finn et al., 2016). NCBI BLAST was used, and manual corrections were then performed to remove alternative events and redundancy. We analyzed the amino acid lengths, molecular weight (MW) and isoelectric points (PI) on the ExPasy website (http://web.expasy.org/protparam/). ZIP proteins from Arabidopsis and rice were aligned using CLUSTAL_X2 program. Then, the NJ phylogenetic tree was constructed using MEGA7 program with 1,000 bootstrap replicates. Evolutionary distances were calculated using the Poisson correction method and are expressed in terms of the number of amino acid substitutions per site. Potential transmembrane domains in each FtZIP protein were identified using the TMHMM program (Krogh et al., 2001; Qian et al., 2019). Conserved motifs of proteins were predicted using the MEME Suite web server (http://meme-suite.org/) and the number of motifs was set as 10, at a width range from 5 to 200 amino acids.

### 2.3 mRNA expression analysis

The expression levels of *FtZIP* transcripts were analyzed using quantitative real-time PCR (qRT-PCR) assay. Total RNA was extracted from the roots (7-d seedlings), stems (10-d seedlings), leaves (10-d seedlings), flowers, fruits and seeds using an RNAprep Pure Plant Kit (Tiangen, Beijing, China). Then, cDNA synthesis was performed in a 20 μl reaction mixture containing 1 μg of total RNA and a mixture of Hifair^®^ cDNA Synthesis Kit (Yeasen, Shanghai, China). The real-time PCR mixture contained 1 μl cDNA, 1 μl forward and reverse primers, and 10 μl 2 x SYBR Green (TaKaRa, Beijing, China). The qRT-PCR was performed using a CFX96 Touch™ Real-Time PCR detection system (Bio-Rad, Hercules, California, CA, USA). All reactions were performed in three triplicates with the following cycling conditions: 95°C for 3 min; 30 cycles each at 95°C for 10 s and 56°C for 30 s, and 72°C for 20 s. The 2^−ΔΔ^Ct method was used for the analysis of qRT-PCR (Livak and Schmittgen, 2001). The housekeeping gene *FtH3* (ID: HM628903) was used as an internal control (Li C et al., 2019). All primers are shown in **Supplementary Table S1**.

### 2.4 Localization of FtZIPs in *N. benthamiana*

To identify the localization of FtZIPs, The coding regions of the two FtZIP representative genes, *FtZIP2/6* (without stop codons) with XhoI cleavage sites were cloned into pEasyGate100 containing a 35S promoter for enhanced green fluorescence protein (EGFP) by homologous recombination. EGFP was linked to the C-terminus of the ZIP protein. The primers are listed in **Supplementary Table S1**. The pEasyGate100-EGFP was used as a control, and mCherry-labeled AtPIP2A was used as a PM marker. The constructs were introduced into the GV3101 strain of Agrobacterium tumefaciens and then were incubated overnight at 28°C. Cells were harvested, resuspended in infiltration buffer (0.2 mM acetosyringone, 10 mM MgCl_2_, and 10 mM MES), and then infiltrated into 4-week-old *N. benthamiana* leaves with a needleless syringe. After 3 d incubation, the green fluorescence was observed in transformed leaf epidermal cells using a confocal laser-scanning microscope (DMI6000B; Leica, Mannheim, Germany). The fluorescence signal was observed at excitation wavelengths of 488 nm or 561 nm and emission wavelengths of 500–572 nm or 605–635 nm. Three or four leaves per time were observed for three biological replicates.

### 2.5 Yeast complementation assay

The cDNA fragments of *FtZIP2*, *FtZIP3*, *FtZIP4*, *FtZIP5*, *FtZIP6*, *FtZIP7*, *FtZIP9*, *FtZIP10*, *FtZIP11* and *FtZIP12* were amplified and cloned into the *p*YES2 vector. Then, the constructed plasmids were transformed into the *zrt1zrt2* yeast mutant ZHY3, *fet3fet4* yeast mutant DEY1453, *smf1* and *ycf1* yeast mutant BY4741, respectively (Fu et al., 2017; Yue et al., 2021). The lithium acetate/PEG transformation method was used for yeast transformation. Galactose as the glycogen. Yeast strain expressing empty vector or FtZIPs were pre-cultured in SD liquid medium lacking Ura at 30°C for16 h. Precultured cells were diluted to an OD_600_ of 1.0, and 5 μL aliquots were spotted onto synthetic complete medium without Uracil (SD-Ura) plates supplemented with or without 1 mM EDTA, 10 μM and 20 μM BPDS, 12 mM EGTA or 40 μM CdCl_2_ as indicated. Plates were incubated 3 days at 30°C and photographed.

## 3 Results

### 3.1 Identification and classification of *ZIP* genes in Tartary buckwheat

**Figure 1:**
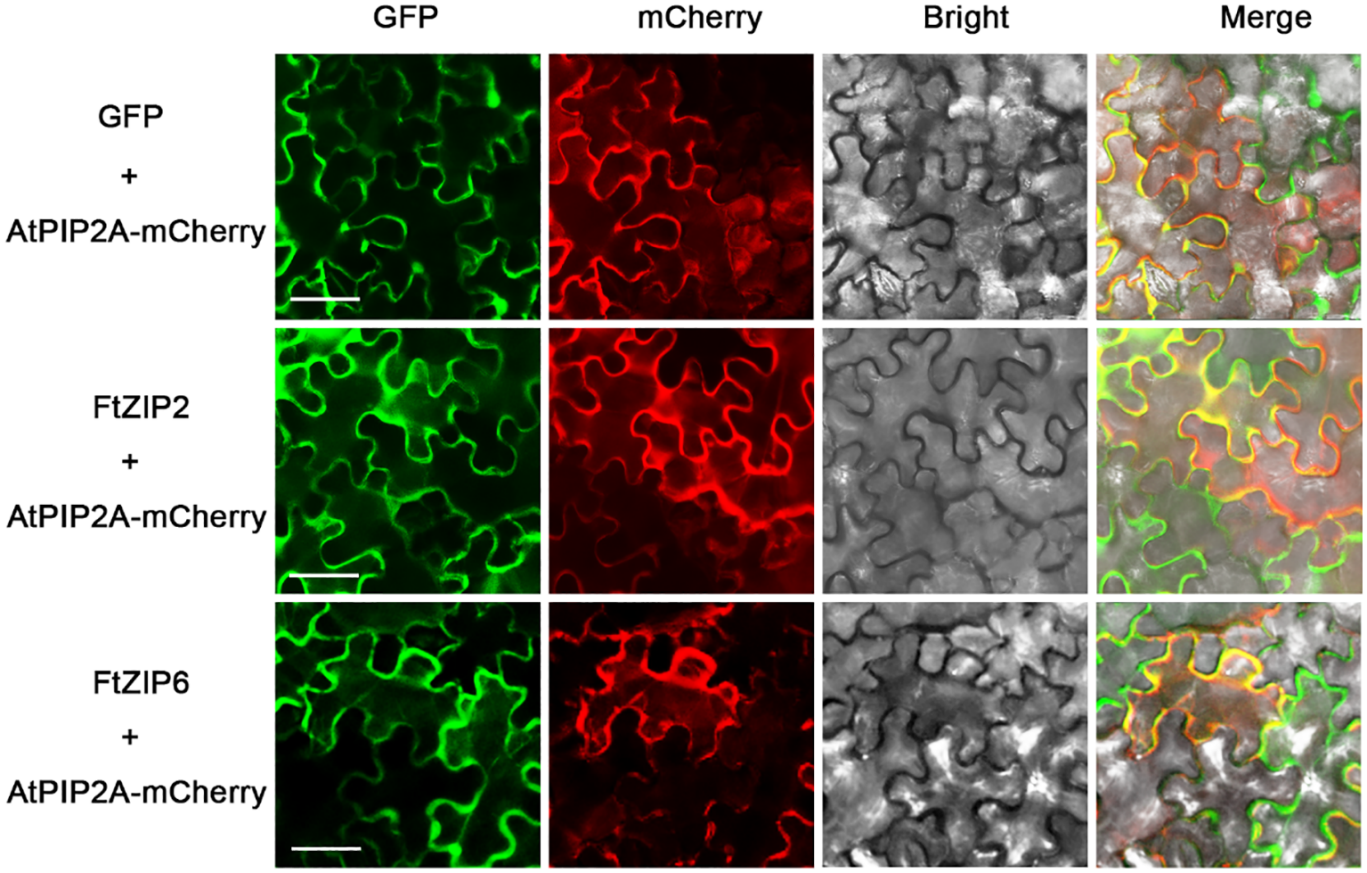
Analysis of the subcellular localization of FtZIP2 and FtZIP6. Confocal images of N. *benthamiana* leaves expressing FtZIP2 and FtZIP6. An mCherry-labeled plasma membrane marker (AtPIP2A) was coexpressed to visualize the plasma membrane. An empty vector was used as a positive control. Bars = 50 μm.

Although the ZIP family has been reported in various species, the genes of this family have not been reported in Tartary buckwheat. In this study, a total of 13 putative ZIP genes were identified from the Tartary buckwheat genome. Here, these *ZIP* genes were provisionally named as FtZIP1 to FtZIP13 (**Table 1**) according to their locations on chromosomes. We found that ZIPs were unevenly distributed on the chromosomes. There were three genes positioned on the second and third, two genes on the first, seventh and eighth, and one gene on the fifth chromosome, but no genes locating on the fourth chromosome, respectively (**Supplementary Figure S1**). The amino acid (aa) length of FtZIPs varied from 200 aa (FtZIP9) to 426 aa (FtZIP1), and the PI ranged from 5.21 (FtZIP2) to 8.10 (FtZIP13) (**Table 1**). In addition, most of FtZIPs had 8 TM domains, except for FtZIP9 (4 TM) and FtZIP11 (7 TM). In addition, most of the FtZIP proteins were predicted to be localized on the plasma membrane (**Table 1**), which is consistent with the known characteristics of the *ZIP* gene (Fu et al., 2017), while FtZIP9 might be also localized in cytoplasm. Next, we checked the localizations of *FtZIP2* and *FtZIP6* in tobacco leaves. The results confirmed that both of them localized in the membrane (**Figure 1**; **Supplementary Figures S2**, **3**), which is in line with the predicted results.

**Table 1:** Localization and physicochemical characteristics of FtZIPs.

Gene name	ID	Subcellular Localization	MW (KDa)	Protein length	PI	TMD	Grand average of hydropathicity
FtZIP1	FtPinG0005816600.01.T01	PlasmaMembrane	45.29	426	5.84	8	0.36
FtZIP2	FtPinG0001701300.01.T01	PlasmaMembrane	42.81	403	5.21	8	0.345
FtZIP3	FtPinG0006268600.01.T01	PlasmaMembrane	37.87	367	5.58	8	0.563
FtZIP4	FtPinG0003390700.01.T01	PlasmaMembrane	41.13	387	7.67	8	0.44
FtZIP5	FtPinG0006885600.01.T01	PlasmaMembrane	36.73	343	6.35	8	0.564
FtZIP6	FtPinG0004012100.01.T01	PlasmaMembrane	37.99	359	6.62	8	0.558
FtZIP7	FtPinG0003990300.01.T01	PlasmaMembrane	39.58	371	6.26	8	0.533
FtZIP8	FtPinG0007900700.01.T01	PlasmaMembrane	34.31	328	6.08	8	0.815
FtZIP9	FtPinG0005140200.01.T01	PlasmaMembrane/Cytoplasmic	21.15	200	6.07	4	0.376
FtZIP10	FtPinG0007555000.01.T01	PlasmaMembrane	36.4	342	6.09	8	0.518
FtZIP11	FtPinG0004751000.01.T01	PlasmaMembrane	29.56	285	5.78	7	0.664
FtZIP12	FtPinG0007186800.01.T01	PlasmaMembrane	32.17	297	7.65	8	0.586
FtZIP13	FtPinG0007186600.01.T01	PlasmaMembrane	38.26	355	8.1	8	0.492

**Figure 2:**
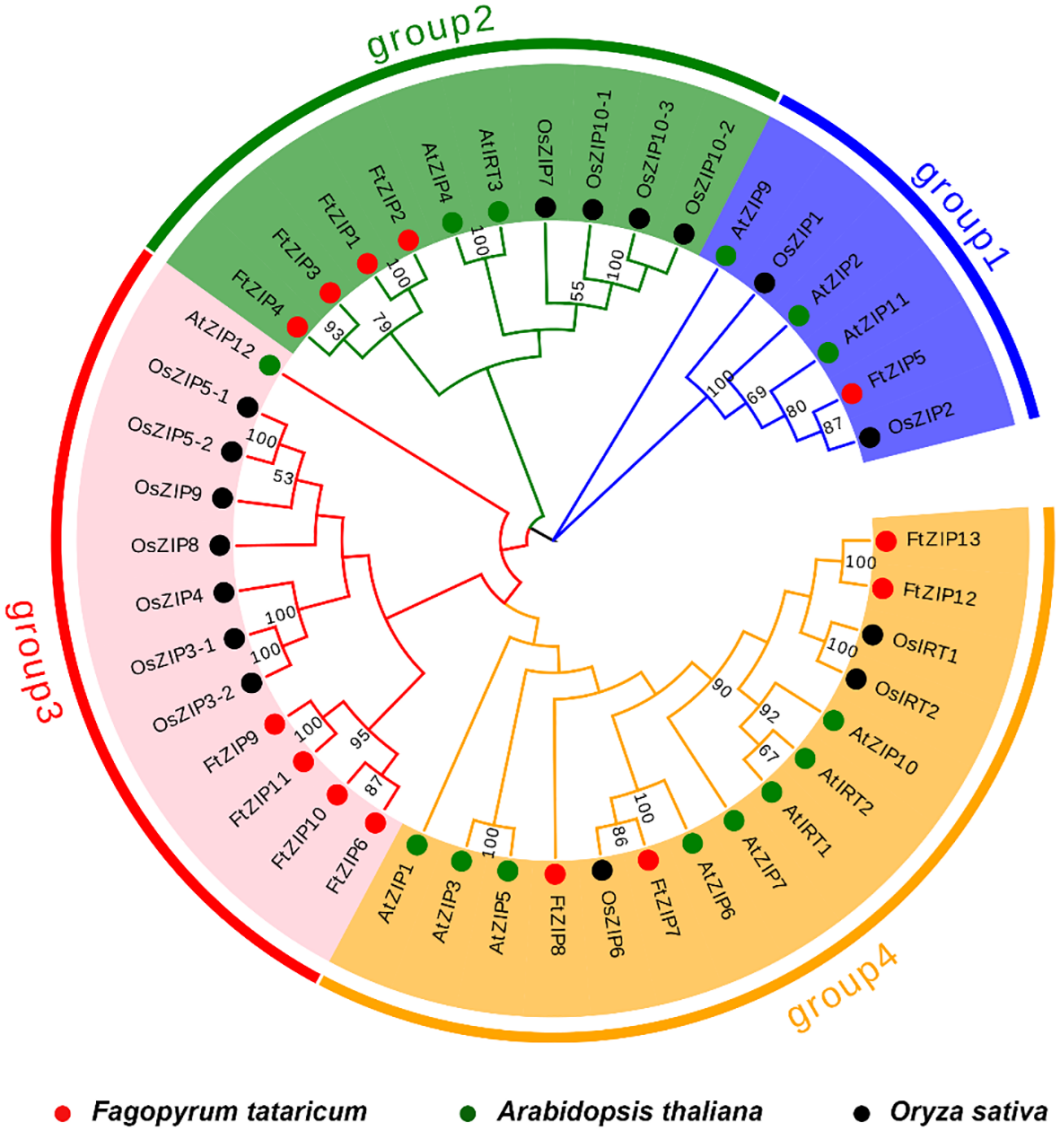
Phylogenetic tree of ZIPs from plants. Based on the full-length protein sequences, the phylogenetic tree was constructed using the neighbor joining method. The plants included *Fagopyrum tatarium* (Ft), *Arabidopsis thaliana* (At) and *Oryza sativa* (Os).The four different groups are indicated by different colors.

We constructed a phylogenetic tree using 13 FtZIPs and 30 ZIPs from rice and Arabidopsis to identify the phylogenetic relationship between FtZIPs and other ZIPs in planta (**Figure 2**). The result showed that these ZIPs could be divided into four groups: group 1, group 2, group 3 and group 4 (**Figure 2**). Groups 2, 3 and 4 contained the most ZIPs, while group 1 contained little AtZIP and OsZIP. The result shows that these ZIPs proteins may have a conserved function in plants. Except for FtZIP5, the other FtZIPs are evenly divided into groups 1, 2 and 3, indicating that FtZIP5 has different functions from other FtZIPs. The ZIP proteins in the same cluster often shared a similar gene structure. Additionally, we found that the FtZIP7 exhibited a close relationship with AtZIP6, indicating that FtZIP7 shared a similar function with AtZIP6, both of which are involved in Zn transportation (Lee et al., 2021).

### 3.2 Domains, motif structure, and gene structure analysis

**Figure 3:**
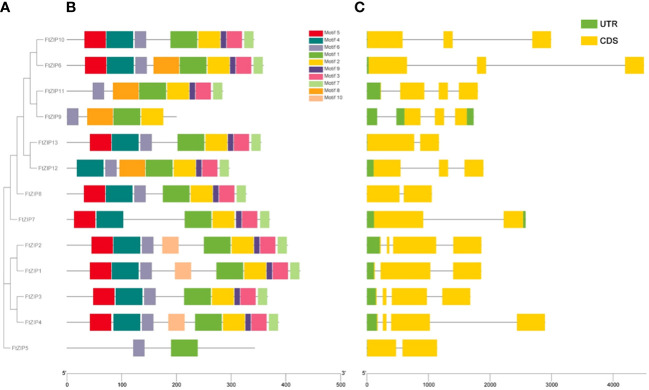
Gene structures and conserved motifs of these identified 13 FtZIP proteins. **(A)** Phylogenetic relationship of these *FtZIP* genes; **(B)** Conserved protein motifs of these FtZIP proteins. The boxes in different colors represent different motifs; and the gray lines represent non-conserved sequences; **(C)** Exon-intron structures of *FtZIP* genes. Green boxes, yellow boxes, and gray lines represent UTRs, exons, and introns, respectively, and their lengths are shown proportionally.

**Figure 4:**
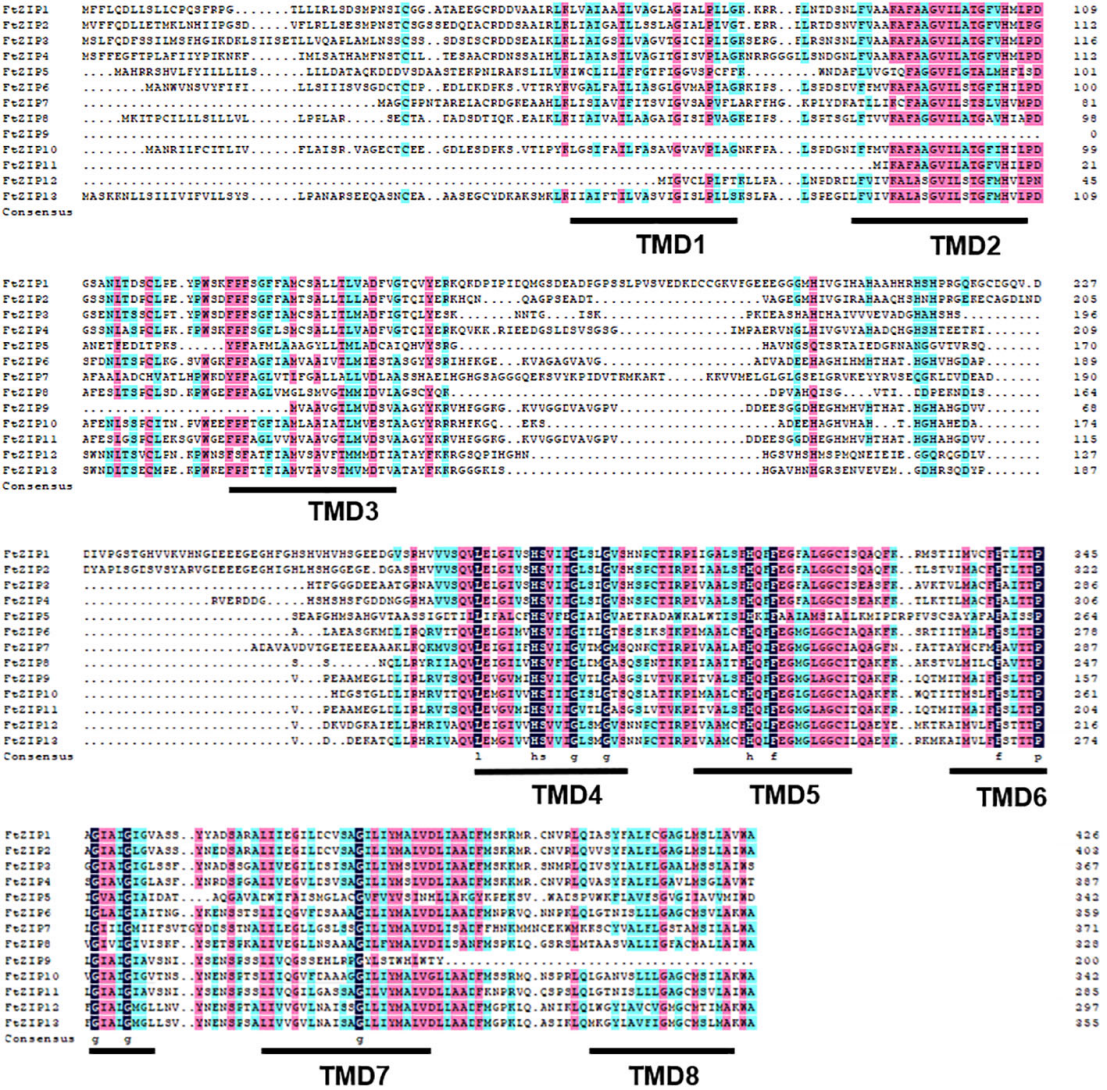
Alignment of FtZIP proteins. FtZIP proteins were aligned using ClustalW. The conserved amino acids are indicated by dark and similar amino acids are shaded with pink color. Transmembrane (TM) domains are shown as lines above the sequences and numbered TM1 to TM8.

To further understand the function of FtZIPs, we analyzed gene structure and conserved motifs. Using the MEME tool, we identified 10 conserved motifs with lengths ranging from 6 to 50 amino acids (**Figure 3B**). The protein structure was different among the members of FtZIPs. The distribution features of the 10 predicted motifs in FtZIPs were in line with the phylogenetic analysis (**Figure 3A**). Most of FtZIPs contained 7-9 motifs, while FtZIP9 had 4 motifs and FtZIP5 had only 2 motifs (**Figure 3B**). The exon–intron structure analysis by the Gene Structure Display Server online program exhibited that the number of introns varied from 1 to 3, and exons ranged from 2 to 4 among 13 *FtZIP* genes (**Figure 3C**). In addition, we observed that the length between TMD3 and TMD4 varied, which is usually related to the binding and transport of metal ions (**Figure 4**). Most of FtZIPs contained various histidine-rich domains (HRDs) such as H(XH)_2_, HXH, H(XH)_5_, H(HXH)_2_, and glycine (G) residue is accompanied by HRDs. According to these identified characteristics of the FtZIPs, we had reliable reasons to believe that they are the ZIP family regulating ion input or transport in Buckwheat.

To test the possible response patterns of the *FtZIP* family genes to various stress treatments, cis-regulating elements including ABA-, auxin-, MeJA-, drought-, and Zn deficiency-responsive elements were analyzed in the promoter region (2000bp) of these genes (**Supplementary Figure S4**). We observed that all *FtZIPs* contained light-responsive elements, suggesting that ZIP proteins may play an important role in cellular reactions as a catalyst for photosynthesis in planta. In addition, most of *FtZIPs* genes could respond to MeJA, except for *FtZIP2*, *FtZIP3*, *FtZIP8* and *FtZIP11* genes. We also found that Zn deficiency response elements in the promoters of *FtZIP1*, *FtZIP2*, *FtZIP6*, *FtZIP8* and *FtZIP10*.

### 3.3 Expression analysis of *FtZIP* genes in various tissues and response to stress treatments

**Figure 5:**
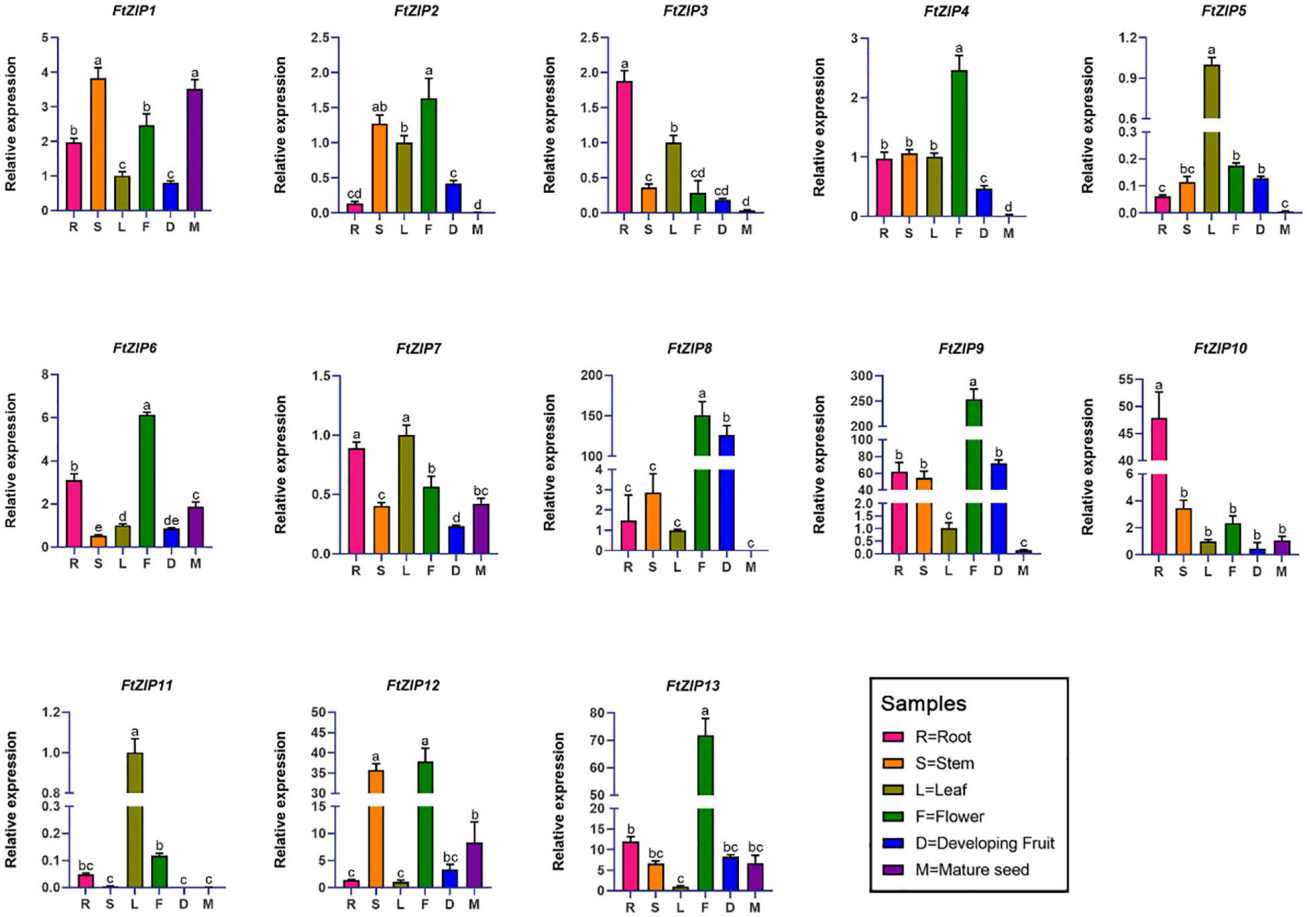
qRT-PCR based relative expression analysis of 13 F*. tataricum ZIP* (*FtZIP*) genes in root, stem, leaf, flower, developing fruit, and mature seed tissues. The relative expression levels of the *FtZIP* genes were normalized by the expression levels of *FtH3*. The expression of *FtZIPs* in leaves were set 1. Values are the means ± SD (n = 3). Statistical significance was determined by ANOVA in combination with post-hoc tests; significant differences (P ≤ 0.05) are indicated by different lowercase letters.

Next, we investigated the expression levels of 13 *FtZIP* genes in the root, stem, leaf, flower, fruit and seed organs, using qRT-PCR assay (**Figure 5**). The results revealed that *FtZIP1*, *FtZIP6* and *FtZIP7* were wide expressions in all indicated tissues. In addition, we found that the transcript of *FtZIP8* in reproductive tissues, including flowerers and fruits, was over 100-fold times compared with that in leaves and *FtZIP4*, *FtZIP6* and *FtZIP13* were specially expressed in flowers. Thus, *FtZIP4*, *FtZIP6*, *FtZIP8* and *FtZIP13* are predicted to play a key role in both flower and fruit development. *FtZIP3* and *FtZIP10* were specially expressed in roots, indicating that they may be involved in ion intake. Notably, only *FtZIP1, FtZIP6, FtZIP12, FtZIP13* had higher level of expression in seeds than in leaves, while others *FtZIPs* were hardly detected.

**Figure 6:**
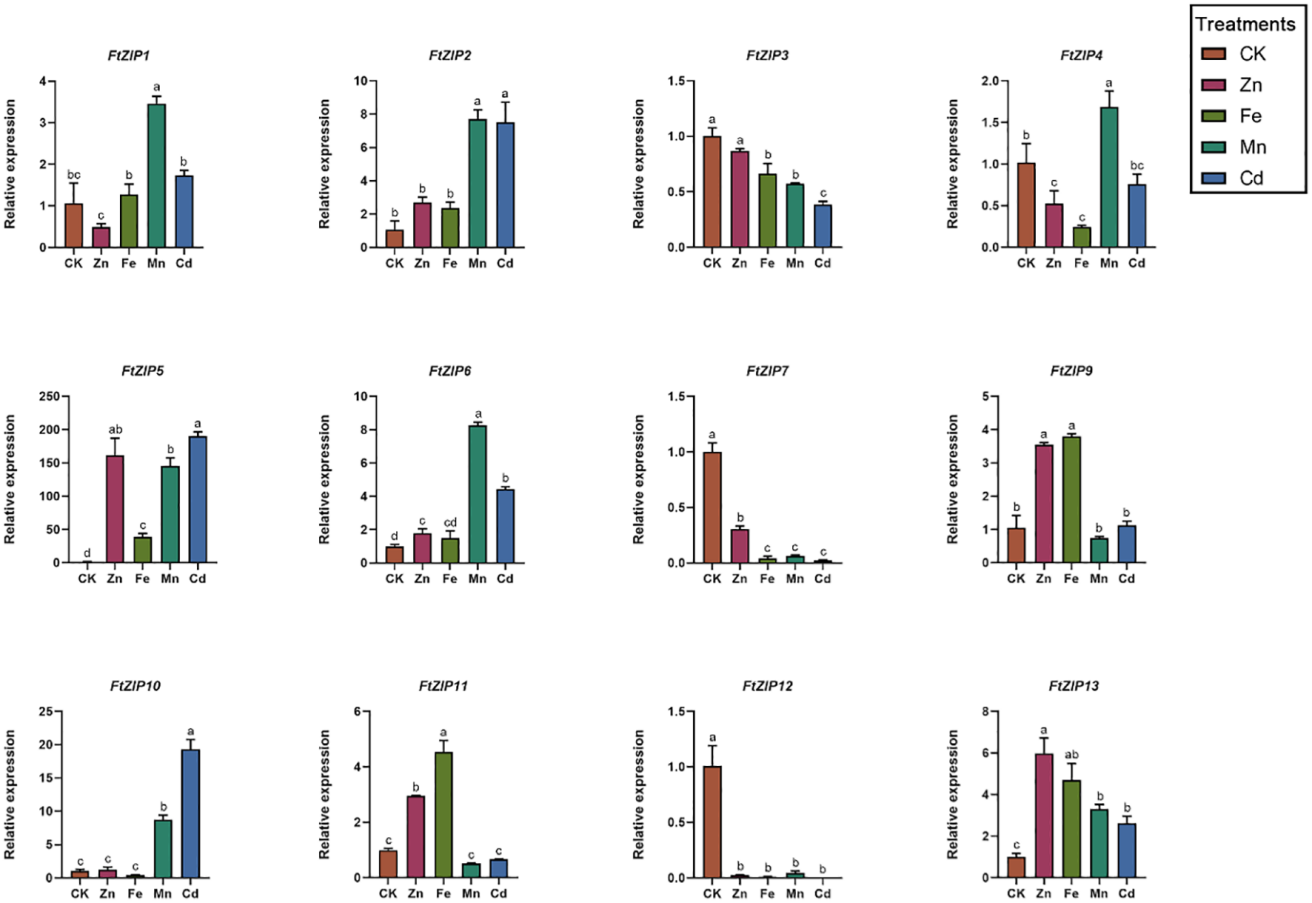
Relative expressions of *FtZIPs* from Tartary buckwheat under various treatments. 21-d seedlings were treated with indicated treatments for 6-h. CK, Hoagland solution; Zn, 75 μM ZnSO_4_ treatment; Cd, 100 μM CdCl_2_; Mn, 100 μM MnCl_2_; Fe,100 μM FeSO_4_. Values are the means ± SD (n = 3). Statistical significance was determined by ANOVA in combination with post-hoc tests; significant differences (P ≤ 0.05) are indicated by different lowercase letters.

Considering that the ZIP gene family has been reported to be involved in transporting zinc, iron and other metallic ions, we also measured the transcriptional levels of *FtZIPs* under ZnSO_4_, FeSO_4_, MnCl_2_ and CdCl_2_ treatments (**Figure 6**). For *FtZIP1*, its relative transcriptional level was also induced by Mn^2+^, with 3-fold increase compared with the normal conditions, while other treatments did not have an effect on its mRNA level. Both of Mn^2+^ and Cd^2+^ could significantly trigger relative expressions of *FtZIP2*, *FtZIP6* and *FtZIP10*. Interestingly, *FtZIP11* was up-regulated by Zn^2+^ and Fe^2+^, while down-regulated by Mn^2+^ and Cd^2+^. In contrast, *FtZIP4* was upregulated by Mn^2+^, while down-regulated by Zn^2+^ and Fe^2+^. For *FtZIP5*, we found that it was highly induced by heavy metals, over 150-fold augment compared with that under the normal conditions. Notably, all treatments markedly repressed the transcripts of *FtZIP3*, *FtZIP7* and *FtZIP12*.

### 3.4 Functional complementation analysis of the FtZIP family in yeast mutants

To identify whether FtZIPs were able to transport metals, the important metal transport proteins, we employed defective metal uptake systems. The *zrt1zrt2* and *fet3fet4* mutants are defective in in both low- and high-affinity Zn and Fe uptake system, respectively (Eide et al., 1996; MacDiarmid et al., 2000). The *smf1* mutant is sensitive to EGTA, a Mn chelator (Cohen et al., 2000; Zhang et al., 2017). The *ycf1* yeast system is defective in pumping Cd into vacuoles (Meng et al., 2017).

**Figure 7:**
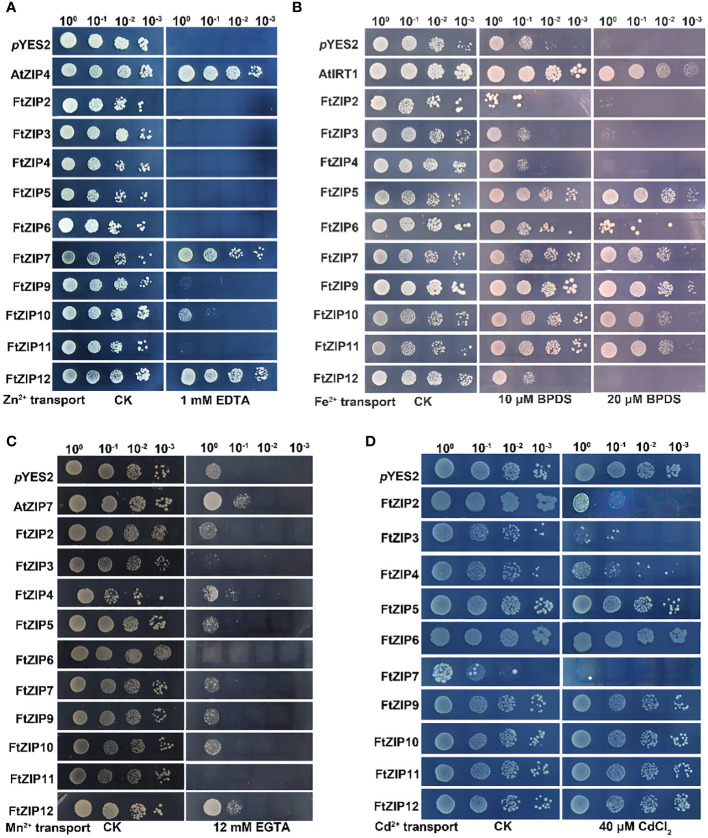
Complementation of yeast metal uptake-defective mutants with FtZIP genes on selective medium. **(A)** *zrt1zrt2* yeast mutant ZHY3 containing empty (*p*YES2, empty vector) or members of the FtZIP family, was grown on SD-Ura medium containing with 0.6 mM ZnSO_4_ (CK), or 1 mM EDTA. **(B)** *fet3fet4* yeast mutant DEY1453 containing empty (*p*YES2, empty vector) or members of the FtZIP family, was grown on SD-Ura medium supplemented with or without BPDS. **(C)** The *smf1* yeast mutants containing empty (*p*YES2, empty vector) or members of the FtZIP family, were grown on SD-Ura medium supplemented with or without 12 mM EGTA. **(D) **The *ycf1* yeast mutant cells transformed with *p*YES2 empty vector containing with or without *FtZIPs*, and grown on SD-Ura solid medium supplemented with or without 40 μM CdCl_2_. Serial dilutions (10 x) of cultures were spotted. Images were taken after 3 days.

We found that the *Δzrt1zrt1* yeast cells expressing of *FtZIP7* and *FtZIP12* displayed well-growth as the positive control (AtZIP4), and expression of *FtZIP10* could also slightly improve the growth of yeast under Zn-deficient conditions, while other members of the FtZIP family could not restore normal growth (**Figure 7A**). These results suggest that FtZIP7 and FtZIP12 are able to complement *zrt1zrt2* mutant and transport Zn. The expression of *FtZIP5/6/7/9/10/11* notably improve the growth of the *Δfet3fet4* mutant yeast on SD-Ura solid medium in presence of 20 μM Fe^2+^ chelating agent, 4,7-diphenyll,10-phenanthroline disulfonic acid (BPDS), while the growth of yeast expressing of *FtZIP2/3/4/12* was similar with the negative control (*p*YES2, the empty vector) (**Figure 7B**). These results suggest that FtZIP5/6/7/9/10/11 are able to complement *fet3fet4* mutant, but FtZIP2/3/4/12 could not. In addition, the *smf1* mutant yeast expressing the FtZIP family were grown on the SD-Ura solid medium supplemented with or without EGTA. The results showed that only expressing *FtZIP12* significantly improved the growth of yeast, in consistent with the positive control AtZIP7, suggesting that FtZIP12 is capable of complementing *smf1* mutant, but other members are not (**Figure 7C**). In the *ycf1* mutant, all transformants were well grown on the SD-Ura solid medium. After 40 μM Cd treatment, cells expressing *FtZIP5*, *FtZIP6*, *FtZIP9*, *FtZIP10*, *FtZIP11* or *FtZIP12* displayed no significant difference with the negative control, while FtZIP4 had slight ability of complementing *ycf1* mutant (**Figure 7D**). However, yeast cells after transformants of FtZIP2, FtZIP3 or FtZIP7 could hardly grow on the SD-Ura medium supplemented with 40 μM Cd, indicating that these proteins could intake of excessive Cd.

## 4 Discussion

Zn transporter proteins, ZIPs regulate Zn homeostasis, which is necessary for all living organisms. Although the ZIP family has been well reported in many species, such as rice and wheat, the ZIP family has not been well studied in F. *tataricum*. Here, we characterized 13 FtZIPs in buckwheat (**Table 1**), equal to the number of ZIPs in rice, but less than that in Arabidopsis. In addition, most of FtZIP proteins contained 8 putative TMs which is consistent with that proposed by Guerinot (Guerinot, 2000).

In this study, we found that FtZIP2 and FtZIP6 localized in the plasma membrane (**Figure 1**; **Supplementary Figures S2**, **3**). Normally, the N. *benthamiana* transient expression system was used to co-express genes of interest with fluorescent organelle markers. However, when using tobacco epidermal cells to study proteins localized to the plasma membrane, it might be challenging to distinguish their location from the cytoplasmic background. The utilization of native species at the endogenous expression level might yield better results. Additionally, we also analyzed the characteristics of FtZIPs, with results showing a high degree of conservation with *Arabidopsis thaliana*, but not homologous to OsZIP (**Figure 2**). The variable residue length between TM3 and TM4 were found in FtZIP proteins, which is predicted to be directed toward the cytoplasmic side of the plasma membrane, and it was rich in histidine residues, thus providing a cytoplasmic metal ion binding site (Eng et al., 1998; Guerinot, 2000; Zeng et al., 2021). However, FtZIP7 only contained one histidine residue and was predicted to be located in cytoplasmic, but had the conserved G residue (**Table 1**; **Figure 4**). Glycine residues near the TM mediate TM packing (Zhang et al., 2017).

Plant ZIP transporters are partially conserved with BbZIP. For example, functional residues His177 and Gly182 actively participate In the metal (Cd/Zn) released from the metal binding site of BbZIP. vast majority Conservative His117 residue found in plant ZIP protein sequence in BbZIP (Ajeesh Krishna et al., 2020). Undoubtedly, we have also discovered these two conserved sites in members of the FtZIP family of Tartary buckwheat. The His177 and Gly182 are involved in the metal release from the metal-binding site of the BbZIP. Similarly, Glu211 and Gly212 are metal-binding residues in BbZIP. Glu211 of BbZIP is conserved in FtZIP proteins expect for FtZIP5 (Ala232), where Glu is replaced by Ala. Additionally, Gly212 of BbZIP is conserved in FtZIP proteins except for FtZIP5 (Ala233), where Gly is replaced by Ala. The metal-binding site residue that G181 is conserved only with FtZIP5 (**Figure 4**). FtZIP5 is closely related to OsZIP2 (**Figure 2**). Previous studies have shown that OsZIP2 is involved in iron absorption (Pradhan et al., 2020). Our yeast experiments have confirmed that FtZIP5 can grow on iron-deficient culture media (**Figure 7**). It is possible that FtZIP5 also plays a role in iron absorption in Tartary buckwheat.

The cis-regulatory elements present in the promoter region have an important role in gene expression regulation since it harbors various signals/factors responsive elements. Here, we found that the FtZIP family contains biotic and abiotic responsive elements, which is in line with the previous reports. In addition, light responsive elements were found in each member of *FtZIP* genes. Studies have revealed that ZIPs are usually involved in a wide range of cellular processes, such as protein synthesis and photosynthesis (Sinclair and Krämer, 2012). Thus, FtZIPs may have respective functions in various stresses and as a catalyst for cellular reactions.

The analysis of the expression profiles indicates that *FtZIP3* and *FtZIP10* were expressed in root (**Figure 5**), which is similar to *NtZIP5B* which is primarily tested in the root to mediate the absorption of Zn directly from the soil solution (Palusińska et al., 2020). In addition, the expressions of *FtZIP4*, *FtZIP6* and *FtZIP13* and were mainly detected in the flowers (**Figure 5**), which is consistent with *VvZIP3* which is mainly transcribed in developing flowers (Gainza-Cortés et al., 2012).

Transition metals such as Zn, Mn, Fe, and Cu can be toxic when present in excess. The ZIP family transports not only Zn, but also other ions, such as Fe, Mn, Cd, and Cu. The qRT-PCR analyses showed that *FtZIP5* and *FtZIP10* were significantly induced by Cd^2+^, with over 150-fold and 15-fold high expressions compared with the control, respectively (**Figure 6**). However, the ability of Cd transport is moderate (**Figure 7**). These data indicate that FtZIPs are evolutionally conserved, while are also divergent, which is consistent with ABA receptors in Arabidopsis (Fuchs et al., 2014). Rice overexpressing *OsIRT1* plants are sensitive to excess Zn and Cd, indicating that OsIRT1 also transports those metals (Lee and An, 2009). Overexpression of *VsRIT1* (root iron transporter 1) in Arabidopsis increases Cd^2+^ accumulation in Arabidopsis seedlings (Zhang et al., 2020). However, *FtZIP* genes were suppressed in seedlings after MnSO_4_ treatment, indicating that a decrease in *FtZIP* expressions abolishes excessive absorption of Mn^2+^ in plants (Zhang et al., 2018).

The complementary abilities of the FtZIP family members vary among the four yeast mutants. FtZIP12 has abilities of complementing *zrt1zrt2* and *smf1* mutant; FtZIP7 and FtZIP10 complements *zrt1zrt2* and *fet3fet4* mutant; FtZIP5, FtZIP6, FtZIP9 and FtZIP11 only can complement *fet3fet4* mutant; FtZIP2, FtZIP3 and FtZIP7 show complementation with *ycf1* mutant (**Figure 7**). One of the subgroups mainly transports Cd and the other transports Fe. The expression of *FtZIP7* is markedly down-regulated by heavy metals (**Figure 6**), thereby inhibiting the preference of transportation, which is in consistent with *BcZIP2* in *Brassica chinensis* (Wu et al., 2021).

## 5 Conclusion

In this study, we have identified and characterized FtZIP family for the first time in the agronomically important plant *F. tataricum*. Our results show that the FtZIP proteins and genes share the conserved structural and organizational features with other plants. Our results predict the possibility that FtZIPs could be targeted for genetic engineering in order to enhance the resistance against various metal stress.

## Data availability statement

The original contributions presented in the study are included in the article/**Supplementary Material**. Further inquiries can be directed to the corresponding author.

## Author contributions

XZ: Writing – review & editing, Data curation, Investigation. JK: Data curation, Formal analysis, Investigation, Writing – review & editing. LY: Investigation, Writing – review & editing. AW: Writing – review & editing, Resources, Supervision. YY: Supervision, Writing – review & editing, Project administration. XL: Writing – review & editing, Writing – original draft. JW: Project administration, Supervision, Writing – original draft, Writing – review & editing.

## Supplementary material

Supplementary Figure 1 Chromosomal distributions of *ZIP* genes in Tartary buckwheat genome.

Supplementary Figure 2 Analysis of the subcellular localization of FtZIP2 and FtZIP6 (**Supplementary Figure S1**).

Supplementary Figure 3 Analysis of the subcellular localization of FtZIP2 and FtZIP6 (**Supplementary Figure S1**).

Supplementary Figure 4 Distributions of putative cis-elements in -2000 bp upstream regions of *FtZIP* genes. TSS, Transcriptional Start Site.

Supplementary Table 1 The primers used in this study.

## References

[B1] Ajeesh Krishna T. P. Maharajan T. Victor Roch G. Ignacimuthu S. Antony Ceasar S. (2020). Structure, function, regulation and phylogenetic relationship of ZIP family transporters of plants. Front. Plant Sci. 11, 662. doi: 10.3389/fpls.2020.00662 32536933 PMC7267038

[B2] Astudillo C. Fernandez A. C. Blair M. W. Cichy K. A. (2013). The Phaseolus vulgaris ZIP gene family: identification, characterization, mapping, and gene expression. Front. Plant Sci. 4, 286. doi: 10.3389/fpls.2013.00286 23908661 PMC3726863

[B3] Barabasz A. Palusińska M. Papierniak A. Kendziorek M. Kozak K. Williams L. E.. (2018). Functional analysis of ntZIP4B and zn status-dependent expression pattern of tobacco ZIP genes. Front. Plant Sci. 9, 1984. doi: 10.3389/fpls.2018.01984 30687374 PMC6335357

[B4] Bari M. A. El-Shehawi A. M. Elseehy M. M. Naheen N. N. Rahman M. M. Kabir A. H. (2021). Molecular characterization and bioinformatics analysis of transporter genes associated with Cd-induced phytotoxicity in rice (*Oryza sativa* L.). Plant Physiol. Biochem. PPB 167, 438–448. doi: 10.1016/j.plaphy.2021.08.024 34411783

[B5] Briat J. F. Lebrun M. (1999). Plant responses to metal toxicity. Comptes rendus l'Academie Des. Sci. Serie III Sci. la vie 322, 43–54. doi: 10.1016/S0764-4469(99)80016-X 10047953

[B6] Cohen A. Nelson H. Nelson N. (2000). The family of SMF metal ion transporters in yeast cells. J. Biol. Chem. 275, 33388–33394. doi: 10.1074/jbc.M004611200 10930410

[B7] Eide D. Broderius M. Fett J. Guerinot M. L. (1996). A novel iron-regulated metal transporter from plants identified by functional expression in yeast. Proc. Natl. Acad. Sci. U.S.A. 93, 5624–5628. doi: 10.1073/pnas.93.11.5624 8643627 PMC39298

[B8] Eng B. H. Guerinot M. L. Eide D. Saier M. H. Jr. (1998). Sequence analyses and phylogenetic characterization of the ZIP family of metal ion transport proteins. J. membrane Biol. 166, 1–7. doi: 10.1007/s002329900442 9784581

[B9] Evens N. P. Buchner P. Williams L. E. Hawkesford M. J. (2017). The role of ZIP transporters and group F bZIP transcription factors in the Zn-deficiency response of wheat (*Triticum aestivum*). Plant J. 92, 291–304. doi: 10.1111/tpj.13655 28771859 PMC5656842

[B10] Fan Y. Jin Y. Ding M. Tang Y. Cheng J. Zhang K.. (2021). The complete chloroplast genome sequences of eight fagopyrum species: insights into genome evolution and phylogenetic relationships. Front. Plant Sci. 12, 799904. doi: 10.3389/fpls.2021.799904 34975990 PMC8715082

[B11] Finn R. D. Coggill P. Eberhardt R. Y. Eddy S. R. Mistry J. Mitchell A. L.. (2016). The Pfam protein families database: towards a more sustainable future. Nucleic Acids Res. 44, D279–D285. doi: 10.1093/nar/gkv1344 26673716 PMC4702930

[B12] Fu X. Z. Zhou X. Xing F. Ling L. L. Chun C. P. Cao L.. (2017). Genome-wide identification, cloning and functional analysis of the zinc/iron-regulated transporter-like protein (ZIP) gene family in trifoliate orange (*Poncirus trifoliata* L. Raf.). Front. Plant Sci. 8, 588. doi: 10.3389/fpls.2017.00588 28469631 PMC5395618

[B13] Fuchs S. Tischer S. V. Wunschel C. Christmann A. Grill E. (2014). Abscisic acid sensor RCAR7/PYL13, specific regulator of protein phosphatase coreceptors. Proc. Natl. Acad. Sci. U.S.A. 111, 5741–5746. doi: 10.1073/pnas.1322085111 24706923 PMC3992651

[B14] Gainza-Cortés F. Pérez-Dïaz R. Pérez-Castro R. Tapia J. Casaretto J. A. González S.. (2012). Characterization of a putative grapevine Zn transporter, VvZIP3, suggests its involvement in early reproductive development in *Vitis vinifera* L. BMC Plant Biol. 12, 111. doi: 10.1186/1471-2229-12-111 22824090 PMC3432002

[B15] Grotz N. Fox T. Connolly E. Park W. Guerinot M. L. Eide D. (1998). Identification of a family of zinc transporter genes from Arabidopsis that respond to zinc deficiency. Proc. Natl. Acad. Sci. U.S.A. 95, 7220–7224. doi: 10.1073/pnas.95.12.7220 9618566 PMC22785

[B16] Grotz N. Guerinot M. L. (2006). Molecular aspects of Cu, Fe and Zn homeostasis in plants. Biochim. Biophys. Acta 1763, 595–608. doi: 10.1016/j.bbamcr.2006.05.014 16857279

[B17] Guerinot M. L. (2000). The ZIP family of metal transporters. Biochim. Biophys. Acta (BBA) - Biomembr. 1465, 190–198. doi: 10.1016/S0005-2736(00)00138-3 10748254

[B18] Ishimaru Y. Suzuki M. Kobayashi T. Takahashi M. Nakanishi H. Mori S.. (2005). OsZIP4, a novel zinc-regulated zinc transporter in rice. J. Exp. Bot. 56, 3207–3214. doi: 10.1093/jxb/eri317 16263903

[B19] Jiang Y. Chen X. Chai S. Sheng H. Sha L. Fan X.. (2021). TpIRT1 from Polish wheat (*Triticum polonicum* L.) enhances the accumulation of Fe, Mn, Co, and Cd in Arabidopsis. Plant Sci. 312, 111058. doi: 10.1016/j.plantsci.2021.111058 34620452

[B20] Kavitha P. G. Kuruvilla S. Mathew M. K. (2015). Functional characterization of a transition metal ion transporter, OsZIP6 from rice (*Oryza sativa* L.). Plant Physiol. Biochem. PPB 97, 165–174. doi: 10.1016/j.plaphy.2015.10.005 26476396

[B21] Krogh A. Larsson B. von Heijne G. Sonnhammer E. L. (2001). Predicting transmembrane protein topology with a hidden Markov model: application to complete genomes. J. Mol. Biol. 305, 567–580. doi: 10.1006/jmbi.2000.4315 11152613

[B22] Lee S. An G. (2009). Over-expression of OsIRT1 leads to increased iron and zinc accumulations in rice. Plant Cell Environ. 32 (4), 418–416. 10.1111/j.1365-3040.2009.01935.x 19183299

[B23] Lee S. Lee J. Ricachenevsky F. K. Punshon T. Tappero R. Salt D. E. (2021). Redundant roles of four ZIP family members in zinc homeostasis and seed development in Arabidopsis thaliana. Plant J. 108 (4), 1162–1173. doi: 10.1111/tpj.15506 34559918 PMC8613002

[B24] Li C. Zhao H. Li M. Yao P. Li Q. Zhao X.. (2019). Validation of reference genes for gene expression studies in tartary buckwheat (*Fagopyrum tataricum* Gaertn.) using quantitative real-time PCR. PeerJ 7, e6522. doi: 10.7717/peerj.6522 30834187 PMC6396815

[B25] Li Q. J. Liu Y. Wang A. H. Chen Q. F. Wang J. M. Peng L.. (2022). Plastome comparison and phylogenomics of Fagopyrum (Polygonaceae): insights into sequence differences between Fagopyrum and its related taxa. BMC Plant Biol. 22, 339. doi: 10.1186/s12870-022-03715-5 35831794 PMC9281083

[B26] Li S. Liu X. Zhou X. Li Y. Yang W. Chen R. (2019). Improving Zinc and Iron accumulation in maize grains using the Zinc and Iron transporter ZmZIP5. Plant Cell Physiol. 60, 2077–2085. doi: 10.1093/pcp/pcz104 31165152

[B27] Li S. Q. Zhang Q. H. (2001). Advances in the development of functional foods from buckwheat. Crit. Rev. Food Sci. Nutr. 41, 451–464. doi: 10.1080/20014091091887 11592684

[B28] Lin H. Yao Y. Sun P. Feng L. Wang S. Ren Y.. (2023). Haplotype-resolved genomes of two buckwheat crops provide insights into their contrasted rutin concentrations and reproductive systems. BMC Biol. 21, 87. doi: 10.1186/s12915-023-01587-1 37069628 PMC10111841

[B29] Livak K. J. Schmittgen T. D. (2001). Analysis of relative gene expression data using real-time quantitative PCR and the 2(-Delta Delta C(T)) Method. Methods (San Diego Calif) 25, 402–408. doi: 10.1006/meth.2001.1262 11846609

[B30] MacDiarmid C. W. Gaither L. A. Eide D. (2000). Zinc transporters that regulate vacuolar zinc storage in *Saccharomyces cerevisiae* . EMBO J. 19, 2845–2855. doi: 10.1093/emboj/19.12.2845 10856230 PMC203372

[B31] Meng J. G. Zhang X. D. Tan S. K. Zhao K. X. Yang Z. M. (2017). Genome-wide identification of Cd-responsive NRAMP transporter genes and analyzing expression of NRAMP 1 mediated by miR167 in Brassica napus. Biometals 30, 917–931. doi: 10.1007/s10534-017-0057-3 28993932

[B32] Mondal T. K. Ganie S. A. Rana M. K. Sharma T. R. (2013). Genome-wide analysis of zinc transporter genes of maize (*Zea mays*). Plant Mol. Biol. Rep. 32, 605–616. doi: 10.1007/s11105-013-0664-2

[B33] Mu S. Yamaji N. Sasaki A. Luo L. Du B. Che J.. (2021). A transporter for delivering zinc to the developing tiller bud and panicle in rice. Plant J. 105, 786–799. doi: 10.1111/tpj.15073 33169459

[B34] Narayanan N. N. Vasconcelos M. W. Grusak M. A. (2007). Expression profiling of *Oryza sativa* metal homeostasis genes in different rice cultivars using a cDNA macroarray. Plant Physiol. Biochem. PPB 45, 277–286. doi: 10.1016/j.plaphy.2007.03.021 17468002

[B35] Noreen S. Rizwan B. Khan M. Farooq S. (2021). Health benefits of buckwheat (*Fagopyrum Esculentum*), potential remedy for diseases, rare to cancer: A Mini Review. Infect. Disord. Drug Targets 21, e170721189478. doi: 10.2174/1871526520999201224122605 33357186

[B36] Palusińska M. Barabasz A. Kozak K. Papierniak A. Maślińska K. Antosiewicz D. (2020). Zn/Cd status-dependent accumulation of Zn and Cd in root parts in tobacco is accompanied by specific expression of ZIP genes. BMC Plant Biol. 20, 1–19. 31969116 10.1186/s12870-020-2255-3 PMC6977228

[B37] Pedas P. Schjoerring J. K. Husted S. (2009). Identification and characterization of zinc-starvation-induced ZIP transporters from barley roots. Plant Physiol. Biochem. PPB 47, 377–383. doi: 10.1016/j.plaphy.2009.01.006 19249224

[B38] Pedas P. Ytting C. K. Fuglsang A. T. Jahn T. P. Schjoerring J. K. Husted S. (2008). Manganese efficiency in barley: identification and characterization of the metal ion transporter HvIRT1. Plant Physiol. 148, 455–466. doi: 10.1104/pp.108.118851 18614714 PMC2528110

[B39] Pradhan S. K. Pandit E. Pawar S. Pradhan A. Behera L. Das S. R.. (2020). Genetic regulation of homeostasis, uptake, bio-fortification and efficiency enhancement of iron in rice. Environ. Exp. Bot. 177, 104066. doi: 10.1016/j.envexpbot.2020.104066

[B40] Qian W. Yang X. Li J. Luo R. Yan X. Pang Q. (2019). Genome-wide characterization and expression analysis of aquaporins in salt cress (*Eutrema salsugineum*). PeerJ 7, e7664. doi: 10.7717/peerj.7664 31565576 PMC6745184

[B41] Sinclair S. A. Krämer U. (2012). The zinc homeostasis network of land plants. Biochim. Biophys. Acta 1823, 1553–1567. doi: 10.1016/j.bbamcr.2012.05.016 22626733

[B42] Sinclair S. A. Senger T. Talke I. N. Cobbett C. S. Haydon M. J. Krämer U. (2018). Systemic upregulation of MTP2- and HMA2-mediated Zn partitioning to the shoot supplements local Zn deficiency responses. Plant Cell 30, 2463–2479. doi: 10.1105/tpc.18.00207 30150315 PMC6241274

[B43] Tan L. Zhu Y. Fan T. Peng C. Wang J. Sun L.. (2019). OsZIP7 functions in xylem loading in roots and inter-vascular transfer in nodes to deliver Zn/Cd to grain in rice. Biochem. Biophys. Res. Commun. 512, 112–118. doi: 10.1016/j.bbrc.2019.03.024 30871778

[B44] Tiong J. McDonald G. K. Genc Y. Pedas P. Hayes J. E. Toubia J.. (2014). HvZIP7 mediates zinc accumulation in barley (*Hordeum vulgare*) at moderately high zinc supply. New Phytol. 201, 131–143. doi: 10.1111/nph.12468 24033183

[B45] Tsunemitsu Y. Genga M. Okada T. Yamaji N. Ma J. F. Miyazaki A.. (2018). A member of cation diffusion facilitator family, MTP11, is required for manganese tolerance and high fertility in rice. Planta 248, 231–241. doi: 10.1007/s00425-018-2890-1 29700611

[B46] Wang X. Zhong F. Woo C. H. Miao Y. Grusak M. A. Zhang X.. (2017). A rapid and efficient method to study the function of crop plant transporters in Arabidopsis. Protoplasma 254, 737–747. doi: 10.1007/s00709-016-0987-6 27240439

[B47] Wong C. K. E. Cobbett C. S. (2009). HMA P-type ATPases are the major mechanism for root-to-shoot Cd translocation in Arabidopsis thaliana. New Phytol. 181, 71–78. doi: 10.1111/j.1469-8137.2008.02638.x 19076718

[B48] Wu X. Su N. Yue X. Fang B. Zou J. Chen Y.. (2021). IRT1 and ZIP2 were involved in exogenous hydrogen-rich water-reduced cadmium accumulation in *Brassica chinensis* and *Arabidopsis thaliana* . J. hazard. mater. 407, 124599. doi: 10.1016/j.jhazmat.2020.124599 33360184

[B49] Yang M. Li Y. Liu Z. Tian J. Liang L. Qiu Y.. (2020). A high activity zinc transporter OsZIP9 mediates zinc uptake in rice. Plant J. 103, 1695–1709. doi: 10.1111/tpj.14855 32449251

[B50] Yue X. Song J. Fang B. Wang L. Zou J. Su N.. (2021). BcNRAMP1 promotes the absorption of cadmium and manganese in Arabidopsis. Chemosphere 283, 131113. doi: 10.1016/j.chemosphere.2021.131113 34146878

[B51] Zeng H. Wu H. Yan F. Yi K. Zhu Y. (2021). Molecular regulation of zinc deficiency responses in plants. J. Plant Physiol. 261, 153419. doi: 10.1016/j.jplph.2021.153419 33915366

[B52] Zhang L. Li X. Ma B. Gao Q. Du H. Han Y.. (2017). The tartary buckwheat genome provides insights into rutin biosynthesis and abiotic stress tolerance. Mol. Plant 10 (9), 1224–1237. doi: 10.1016/j.molp.2017.08.013 28866080

[B53] Zhang T. Liu J. Fellner M. Zhang C. Sui D. Hu J. (2017). Crystal structures of a ZIP zinc transporter reveal a binuclear metal center in the transport pathway. Sci. Adv. 3, e1700344. doi: 10.1126/sciadv.1700344 28875161 PMC5573306

[B54] Zhang X. Li X. Tang L. Peng Y. Qian M. Guo Y.. (2020). The root iron transporter 1 governs cadmium uptake in *Vicia sativa* roots. J. hazard. mater. 398, 122873. doi: 10.1016/j.jhazmat.2020.122873 32768815

[B55] Zhang X. D. Meng J. G. Zhao K. X. Chen X. Yang Z. M. (2018). Annotation and characterization of Cd-responsive metal transporter genes in rapeseed (*Brassica napus*). Biometals 31, 107–121. doi: 10.1007/s10534-017-0072-4 29250721

[B56] Zhang X. Y. Zhang X. Zhang Q. Pan X. X. Yan L. C. Ma X. J.. (2017). Zea mays Fe deficiency-related 4 (ZmFDR4) functions as an iron transporter in the plastids of monocots. Plant J. 90, 147–163. doi: 10.1111/tpj.13482 28103409

