# Supplementary figures and images for: Functional characterization of Fagopyrum tataricum ZIP gene family as a metal ion transporter
Source: Front Plant Sci. 2024 Apr 12;15:1373066. doi: 10.3389/fpls.2024.1373066 (PMC11062324; doi:10.3389/fpls.2024.1373066)

## Slide 1
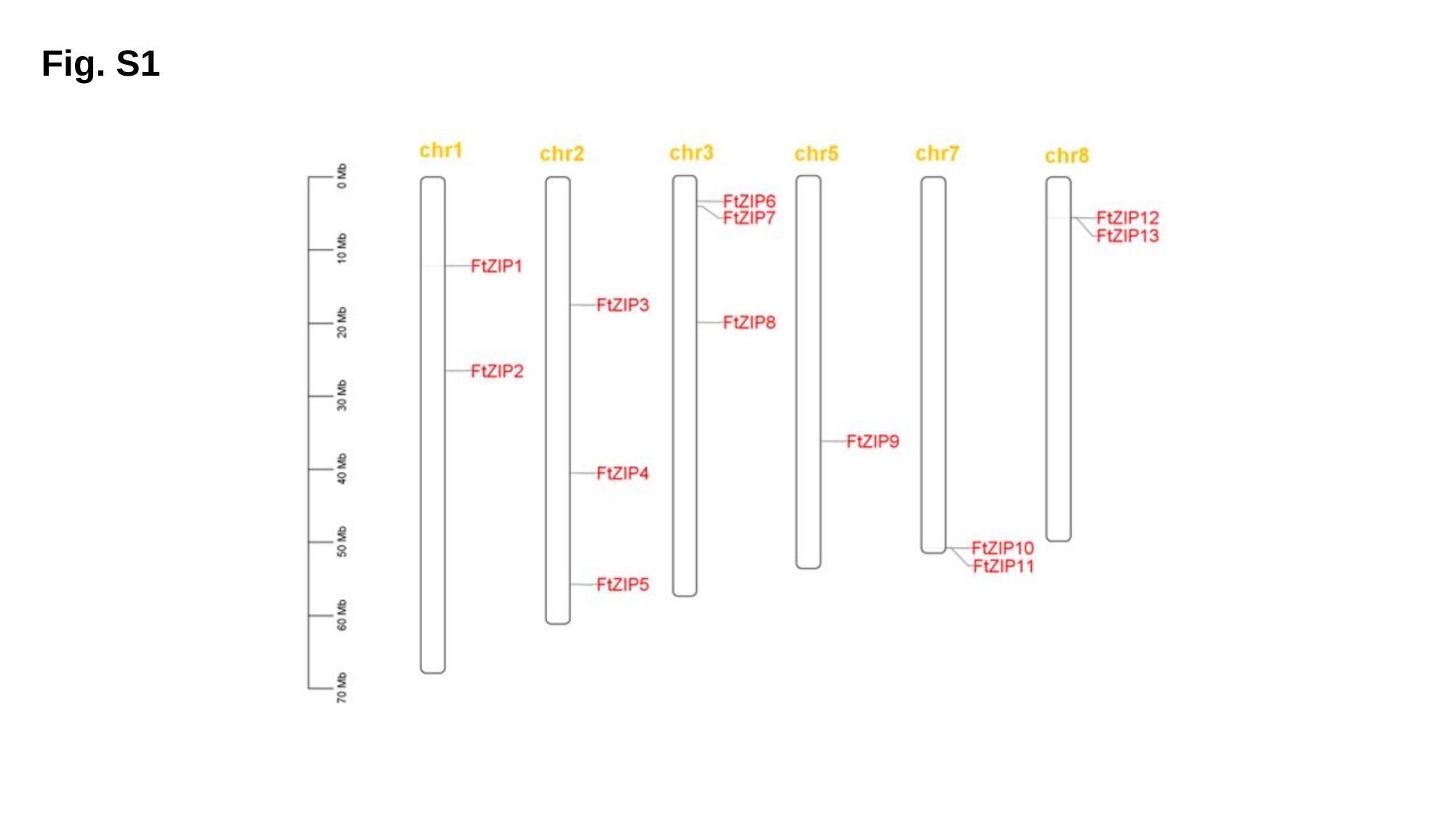

Fig. S1

## Slide 2
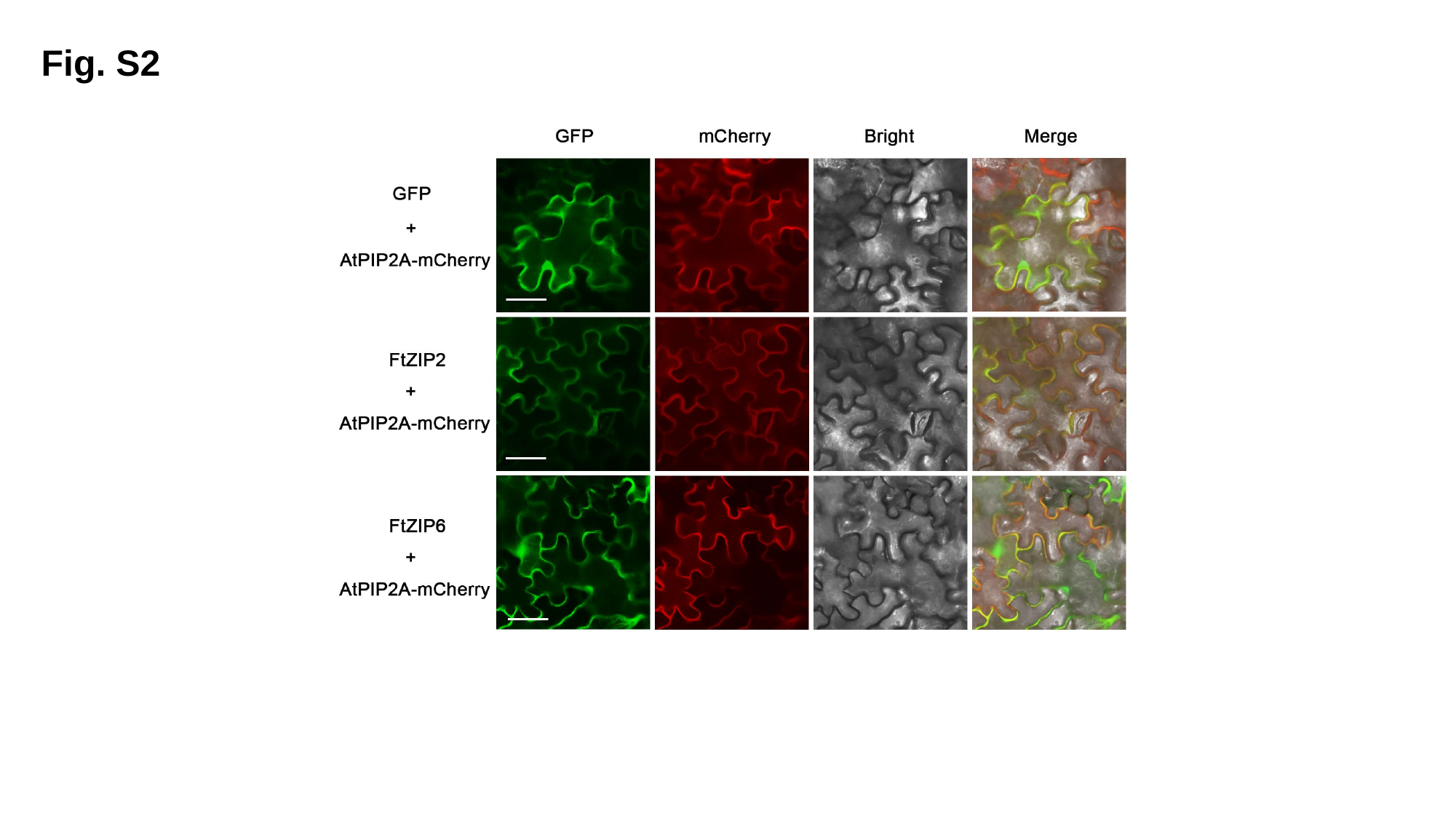

Fig. S2

## Slide 3
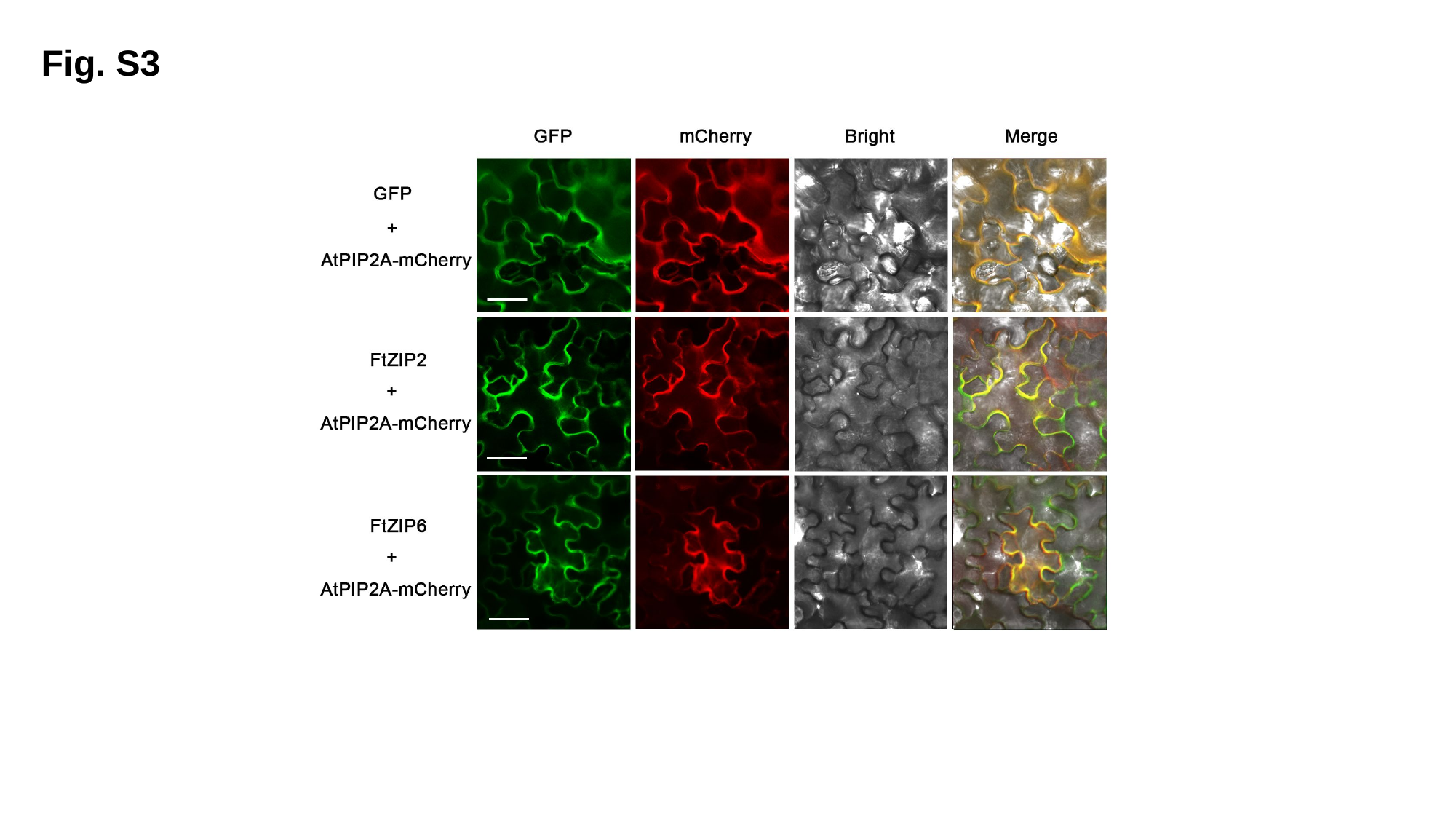

Fig. S3

## Slide 4
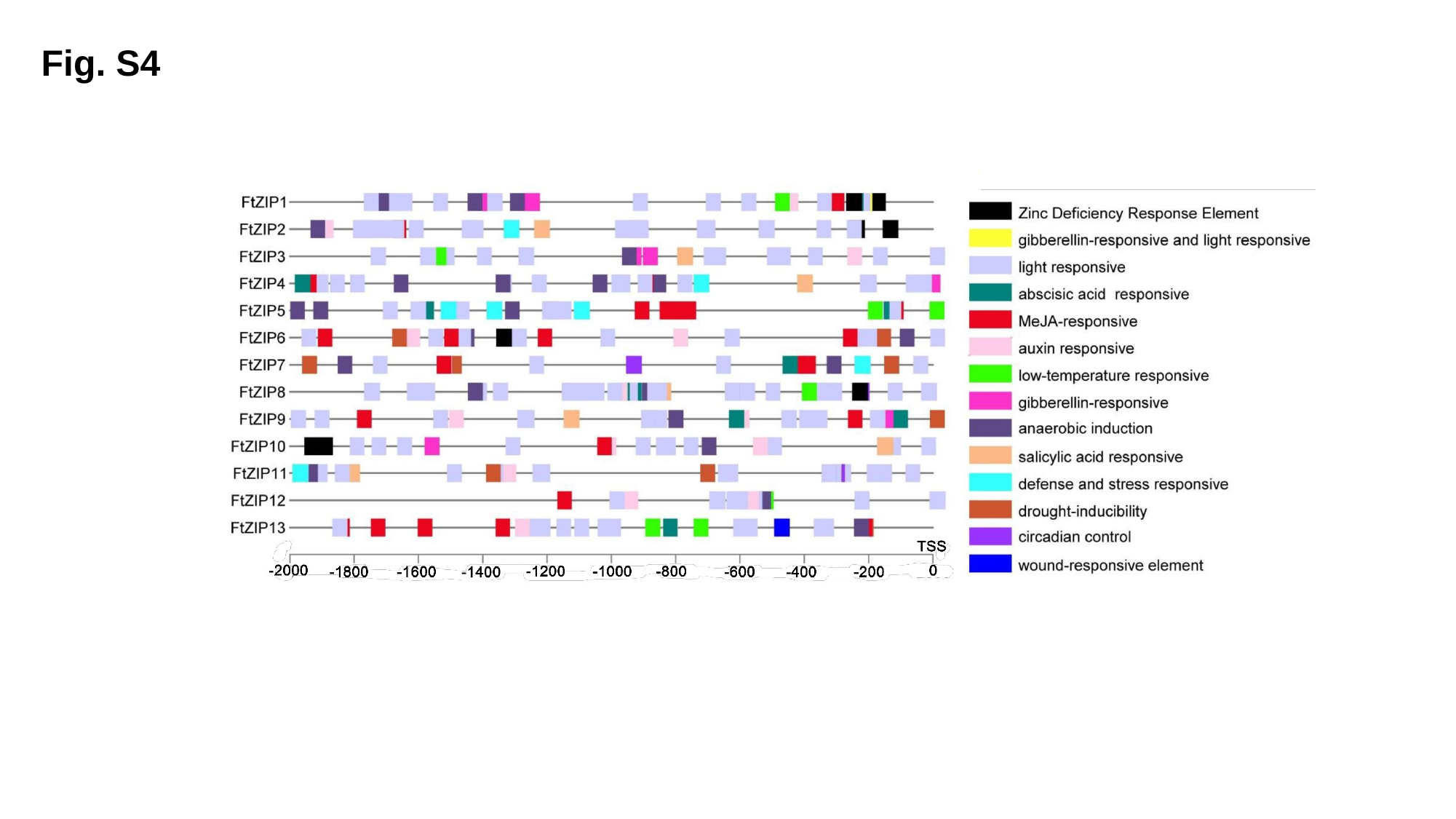

Fig. S4

Supplement: Supplementary Figure 1 — Chromosomal distributions of ZIP genes in Tartary buckwheat genome. [file Presentation_1.pptx]
